# Supplementary material for: Machine Learning Approaches for Activity Recognition and/or Activity Prediction in Locomotion Assistive Devices—A Systematic Review
Source: Sensors (Basel). 2020 Nov 6;20(21):6345. doi: 10.3390/s20216345 (PMC7664393; doi:10.3390/s20216345)
Supplement: Supplementary file 1 [file sensors-20-06345-s001.pdf]

sensors

Supplementary Material - 1

1. Detailed search strategy

The systematic review is reported in accordance with Preferred Reporting Items for Systematic Reviews and Meta Analyses (PRISMA) guidelines [1]. The search strategies of the three selected databases are detailed in this section.

1.1. PubMed

1.1.1. Search equation

In PubMed, the following search equation was used:
((((exoskeleton OR exoskeleton robot OR powered exoskeleton OR powered lower limb exoskeleton OR wearable exoskeleton OR lower limb exoskeleton OR lower limb prostheses OR lower limb prosthesis OR transfemoral amputee OR powered prosthesis OR above-knee amputation OR powered lower limb prosthesis control OR powered above-knee prosthesis OR transtibial amputation OR prosthesis use OR powered prosthesis leg OR amputees OR orthotics OR orthoses OR orthoses OR orthotics)) AND (intent recognition OR locomotion mode classification OR terrain recognition system OR user intent recognition OR locomotion mode recognition OR pattern recognition OR user-independent intent recognition OR terrain recognition OR terrain-adaptive system OR adaptive pattern classifier OR human motion intent)))

1.1.2. Additional filters

The following articles types were selected:
Case reports, classical article, clinical study, clinical trial, comparative study, controlled clinical trial, evaluation studies, journal article, letter, multicenter study, pragmatic clinical trial, randomized controlled trial, patents and conference papers.
Only studies on humans were included.

1.2. Web of Science

1.2.1. Search equation

In Web of Science, the following search equation was used:
(TS = ((Exoskeleton OR Exoskeleton robot OR Powered exoskeleton OR Powered lower limb exoskeleton OR wearable exoskeleton OR lower limb exoskeleton OR Lower limb prostheses OR lower limb prosthesis OR Transfemoral amputee OR powered prosthesis OR Above-knee amputation OR Powered Lower Limb Prosthesis Control OR powered above-knee prosthesis OR transtibial amputation OR prosthesis use OR powered prosthesis leg OR amputees OR Orthotics OR orthoses OR orthosis OR orthotics) AND (Intent Recognition OR Locomotion Mode Classification OR Terrain recognition system OR user intent recognition OR Locomotion mode recognition OR Pattern Recognition OR User-Independent Intent Recognition OR terrain recognition OR terrain-adaptive system OR adaptive pattern classifier OR human motion intent))) AND LANGUAGE: (English) AND DOCUMENT TYPES: (Article OR Data Paper OR Letter OR Proceedings Paper)

sensors

MDPI

Supplementary Material 2

1. Quality assessment of the studies

1.1. Criteria used for scoring the studies

Based on the QualSyst Tool [2], the following twelve criteria were used to assess the quality of the studies:

- Criteria 1: Question/Objective sufficiently described?
- Criteria 2: Study design evident and appropriate?
- Criteria 3: Subject characteristics sufficiently described and representative?
- Criteria 4: Experimental protocol sufficiently described?
- Criteria 5: Critical Timing Provided?
- Criteria 6: Filtering method sufficiently described?
- Criteria 7: Window length clearly mentioned?
- Criteria 8: Input features clearly mentioned?
- Criteria 9: Machine Learning algorithm clearly mentioned?
- Criteria 10: Evaluation method of the machine learning algorithm clearly mentioned?
- Criteria 11: Results reported with enough detail?
- Criteria 12: Conclusions supported by the results?

Each criterion was evaluated with a score between 0 and 2: 2 indicates “yes”, 1 indicates “partial” and 0 indicates “no”. Additionally, prior to assessing the quality of the studies, the following guidelines were created to ensure consistency in ratings.

sensors

MDPI

Criterion	"YES" = 2	"Partial" = 1	"No" = 0
**C1: Question Objective**	The question and the objective of the study are clearly mentioned.	The question and the objective of the study seems not clear	The question and the objective of the study are not provided.
**C2: Study design**	The study design is appropriated to the question/objective		The study design is not appropriated to the question/objective.
**C3: Subjects characteristics**	The following parameters are given:<br>• Healthy volunteers: number of volunteers, gender, mean and SD for the age, height and weight.<br>• Otherwise: number of volunteers, gender, inclusion/exclusion criteria, mean and SD for the age, height and weight.	The following parameters are given:<br>• Healthy volunteers: number of volunteers, gender, mean without SD for the age, height and weight.<br>• Otherwise (2 options):<br>○ number of volunteers, gender, inclusion/exclusion criteria and mean without SD for the age, height, weight<br>○ number of volunteers, gender, mean and SD for the age, height and weight. Inclusion/exclusion criteria are not given.	Data are missing compared to "Partial".
**C4: Experimental protocol**	The following parameters are given:<br>• Studied locomotion tasks<br>• Walking speed<br>• Transitioning leg (if applicable)<br>• Number of trials / locomotion task	The following parameters are given:<br>• Studied locomotion tasks<br>One of the following parameters are given:<br>• Walking speed<br>• Transitioning leg (if applicable)<br>• Number of trials / locomotion task	More parameters are missing compared to partial.
**C5: Critical Timing**	The critical timings for each transition are given (if applicable).	The critical timings are given but precisely for each transition (e.g. critical timing occurred at foot contact on the new locomotion mode or at foot off of the previous locomotion mode) (if applicable)	The critical timings are not provided, even though the transitions are studied.

sensors

MDPI

Criterion	"YES" = 2	"Partial" = 1	"No" = 0
**C6: Filter**	The filters implemented for each signal are given with the corresponding parameters (e.g. Low-pass 4th order Butterworth filter with a 10 Hz cutoff frequency).	The filters implemented for at least one signal are given with the corresponding parameters.<br>Or the filters implemented for all signals are given without the corresponding parameters (e.g. cutoff frequency)	The filters of the signals are not provided.
**C7: Analysis windows**	For each analysis window, the following information are provided:<br>• Beginning and end of each window<br>• Beginning or end of each window and window length.<br>• If multiple windows or sliding windows are used, the overlap or the window increment is provided	One information is not provided (window length or window increment or overlap or beginning or end of each analysis window). For instance, the beginning of the window is provided but the end or window length are not provided.	No information concerning analysis window are given.
**C8: Features**	The feature set is clearly defined. The equations of each feature are provided or given with references.	The feature set is clearly defined but features equations are not given (no references). Or the equations are given but a feature reduction technique is used but the final feature set is not explicitly provided (for instance PCA to reduce the size of the feature set, but the final number of features is not given).	The extracted features are not mentioned.<br><br>Note that if the raw data of the sensors were fed into the Machine Algorithm, the criterion was rated 2 out of 2.
**C9: Algorithms**	The tested algorithms are clearly mentioned, the parameters of each algorithm are provided.	The tested algorithms are mentioned.	The tested algorithms are not mentioned.
**C10: Evaluation**	The evaluation process of each algorithm is provided (e.g. K-fold cross validation with K = 4)	The evaluation process is given but the parameters are not given (e.g. K not provided for K-fold cross validation). As a result, the data split between train/dev/test sets is unclear.	The evaluation process is not given.

sensors

MDPI

Criterion	"YES" = 2	"Partial" = 1	"No" = 0
**C11: Results**	The results for each algorithm are given (mean and standard deviation).	The results for each algorithm are given without the standard deviation.	The mean and the standard deviation are not given.<br>Or the mean and the standard deviation are given but the results are not provided for one of the tested algorithms.
**C12: Conclusion**	The conclusion is supported by the results		The conclusion is not supported by the results.<br><br>Note that if the results were rated 0 out of 2, the conclusion can still be supported by the results. For instance, the accuracy of the tested algorithms was estimated from graphics readings and the conclusion is supported by those estimations (higher/lower performances).

1.2. *Detailed quality scores of the included studies*

Article	1	2	3	4	5	6	7	8	9	10	11	12	Quality Score
Ai et al. 2017 [3]	2	2	0	0	0	2	2	2	2	2	1	2	70,8%
Beil et al. 2018 [4]	2	2	2	2		2	2	2	2	2	1	1	90,9%
Chen et al. 2013 [5]	0	2	1	1		0	2	2	2	2	2	2	72,7%
Chen et al. 2014 [6]	0	2	1	1	2	2	2	2	2	2	1	2	79,2%
Chen et al. 2015 [7]	0	2	1	2		2	2	2	2	2	1	1	77,3%
Du et al. 2012 [8]	0	2	1	1	2	2	2	2	2	2	0	2	75,0%
Du et al. 2013 [9]	0	2	0	0	0	0	2	2	1	2	0	2	45,8%
Feng et al. 2019 [10]	0	2	1	1		2	2	2	2	1	2	2	77,3%
Godiyal et al. 2018 [11]	0	2	1	2		2	2	2	2	2	2	2	86,4%
Gong et al. 2018 [12]	0	2	2	2		2	2	2	1	2	2	2	86,4%
Gong et al. 2020 [13]	2	2	2	1		2	2	2	1	1	2	2	86,4%
Hernandez et al. 2012 [14]	0	2	0	0	0	0	2	1	1	0	1	2	37,5%
Hernandez et al. 2013 [15]	0	2	0	0	2	0	2	2	2	2	1	0	54,2%
Huang et al. 2009 [16]	2	2	0	2		2	2	2	1	2	1	2	81,8%
Huang et al. 2010 [17]	2	2	0	1	2	2	2	2	2	2	0	2	79,2%
Huang et al. 2011 [18]	2	2	1	1	2	2	2	2	1	2	1	2	83,3%
Kim et al. 2017 [19]	0	2	2	1		1	1	1	2	1	1	2	63,6%
Liu et al. 2016 [20]	0	2	1	2	2	0	2	2	2	2	0	2	70,8%
Liu et al. 2017 [21]	2	2	1	0	0	2	2	2	1	2	0	2	66,7%
Liu et al. 2017 [22]	0	2	0	1		2	1	2	2	1	1	2	63,6%
Long et al. 2016 [23]	2	2	2	1	2	1	0	2	2	2	2	2	83,3%
Mai et al. 2011 [24]	0	2	0	1		1	2	1	1	0	1	2	50,0%
Mai et al. 2018a [25]	0	2	0	0	0	0	2	2	1	1	1	2	45,8%
Mai et al. 2018b [26]	2	2	0	0	0	0	2	1	1	2	1	2	54,2%
Miller et al. 2013 [27]	2	2	2	1		2	2	2	2	2	1	2	90,9%
Moon et al. 2019 [28]	0	2	1	0	0	0	0	2	1	0	0	2	33,3%
Pew et al. 2017 [29]	2	2	1	2	1	2	0	0	1	2	1	2	66,7%
Shell et al. 2018 [30]	2	2	1	0	0	2	2	2	2	2	1	1	70,8%
Simon et al. 2017 [31]	2	2	0	2	1	0	2	2		1	2	2	66,7%
Spanias et al. 2014 [32]	0	2	0	0	0	1	2	2	2	2	0	2	54,2%
Spanias et al. 2015 [33]	0	2	0	1	0	1	1	2	2	2	0	2	54,2%
Spanias et al. 2016a [34]	2	2	0	0	0	1	2	2	2	2	0	2	62,5%
Spanias et al. 2016b [35]	2	2	0	0	0	0	1	2	2	2	1	2	58,3%

Article	1	2	3	4	5	6	7	8	9	10	11	12	Quality Score
Spanias et al. 2017 [36]	2	2	0	0	0	0	1	2	2	2	1	2	58,3%
Spanias et al. 2018 [37]	0	2	0	0	2	0	2	2	2	2	1	2	62,5%
Stolyarov et al. 2017 [38]	2	2	1	1	0	2	2	2	2	2	1	2	79,2%
Su et al. 2019 [39]	0	2	0	2		1	2	2	2	2	2	2	77,3%
Tkach et al. 2013 [40]	0	2	0	2	0	2	2	2	2	2	0	1	62,5%
Wang et al. 2013 [41]	2	2	1	2	0	0	2	2	1	2	1	1	66,7%
Wang et al. 2018 [42]	0	2	0	2	2	2	1	2	2	2	2	2	79,2%
Woodward et al. 2016 [43]	2	2	1	2	2	1	2	2	2	2	2	2	91,7%
Xu et al. 2018 [44]	2	2	1	1	2	0	2	2	1	2	1	2	75,0%
Young et al. 2013a [45]	2	2	0	2	1	1	1	2	2	2	0	1	66,7%
Young et al. 2013b [46]	2	2	0	2	2	1	2	2	2	2	1	1	79,2%
Young et al. 2013c [47]	0	2	1	0	1	0	2	2	2	2	1	2	62,5%
Young et al. 2014a [48]	0	2	0	2	2	1	2	2	2	2	0	1	66,7%
Young et al. 2014b [49]	0	2	0	2	2	1	2	2	2	2	1	2	75,0%
Young et al. 2016 [50]	2	2	1	2	0	1	2	2	2	2	1	1	75,0%
Zhang et al. 2011 [51]	2	2	0	0	2	2	2	2	2	2	0	1	70,8%
Zhang et al. 2013 [52]	2	2	2	2	1	1	2	2	1	0	0	1	66,7%
Zhang et al. 2019 [53]	0	2	1	0		0	1	2	2	2	2	2	63,6%
Zhang et al. 2019 [54]	0	2	1	0		0	1	2	2	2	1	2	59,1%
Zhang et al. 2012 [55]	2	2	0	0	1	0	2	2	2	2	1	1	62,5%
Zheng et al. 2013 [56]	2	2	1	2		1	2	2	2	2	1	2	86,4%
Zheng et al. 2014 [57]	2	2	1	2		1	2	2	2	2	1	2	86,4%
Zheng et al. 2016 [58]	0	2	2	2	0	2	2	2	2	2	1	1	75,0%
Zheng et al. 2019 [59]	0	2	1	1	0	1	2	2	1	1	1	1	54,2%
Zhou et al. 2019 [60]	0	2	0	1	2	0	2	2	1	1	1	1	54,2%

sensors

MDPI

Supplementary Material - 3

1. EMG used in the studies

The EMG recorded in each study are reported in the following table. NP = Not Provided (EMG were used but the locations were not provided). The cell is 1 if the channel is used or empty otherwise.

Article	Semitendinosus	Biceps Femoris	Tensor Fasciae Latae	Rectus Femoris	Vastus Lateralis	Vastus Medialis	Sartorius	Adductor Magnus	Gracilis	Biceps Femoris Long Head	Biceps Femoris Short Head	Ground Electrode	Gluteal Maximus	Gluteal Medius	Iliotibial tract	Medial Gastrocnemius	Tibialis Anterior	Peroneus Longus	Gastrocnemius Lateralis
**Ai et al. 2017 [3]**	NP																		
**Beil et al. 2018 [4]**																			
**Chen et al. 2013 [5]**																			
**Chen et al. 2014 [6]**																			
**Chen et al. 2015 [7]**																			
**Du et al. 2012 [8]**				1	1	1	1	1		1	1		1	1					
**Du et al. 2013 [9]**	1			1	1	1	1			1	1								
**Feng et al. 2019 [10]**																			
**Godiyal et al. 2018 [11]**	1	1		1	1	1		1							1				
**Gong et al. 2018 [12]**																			
**Gong et al. 2020 [13]**																			
**Hernandez et al. 2012 [14]**	1			1	1	1		1		1	1								
**Hernandez et al. 2013 [15]**	1			1	1	1		1		1	1								

Article	Semitendinosus	Biceps Femoris	Tensor Fasciae Latae	Rectus Femoris	Vastus Lateralis	Vastus Medialis	Sartorius	Adductor Magnus	Gracilis	Biceps Femoris Long Head	Biceps Femoris Short Head	Ground Electrode	Gluteal Maximus	Gluteal Medius	Iliotibial tract	Medial Gastrocnemius	Tibialis Anterior	Peroneus Longus	Gastrocnemius Lateralis
Huang et al. 2009 [16]	1			1	1	1	1		1	1	1		1	1					
Huang et al. 2010 [17]	1			1	1	1	1	1	1	1	1		1	1					
Huang et al. 2011 [18]	1			1	1	1	1	1	1	1	1		1	1					
Kim et al. 2017 [19]																			
Liu et al. 2016 [20]	1		1	1	1	1		1	1	1									
Liu et al. 2017 [21]	1		1	1	1	1				1	1								
Liu et al. 2017 [22]																			
Long et al. 2016 [23]																			
Mai et al. 2011 [24]																			
Mai et al. 2018a [25]																			
Mai et al. 2018b [26]																			
Miller et al. 2013 [27]		1			1											1	1		
Moon et al. 2019 [28]																			
Pew et al. 2017 [29]																			
Shell et al. 2018 [30]																			
Simon et al. 2017 [31]																			
Spanias et al. 2014 [32]	1	1	1	1	1	1	1	1	1										
Spanias et al. 2015 [33]	1		1	1				1											

sensors

Article	Semitendinosus	Biceps Femoris	Tensor Fasciae Latae	Rectus Femoris	Vastus Lateralis	Vastus Medialis	Sartorius	Adductor Magnus	Gracilis	Biceps Femoris Long Head	Biceps Femoris Short Head	Ground Electrode	Gluteal Maximus	Gluteal Medius	Iliotibial tract	Medial Gastrocnemius	Tibialis Anterior	Peroneus Longus	Gastrocnemius Lateralis
Spanias et al. 2016a [34]	1	1	1	1	1	1	1	1	1										
Spanias et al. 2016b [35]																			
Spanias et al. 2017 [36]																			
Spanias et al. 2018 [37]	1		1	1				1											
Stolyarov et al. 2017 [38]																			
Su et al. 2019 [39]																			
Tkach et al. 2013 [40]																1	1	1	1
Wang et al. 2013 [41]																			
Wang et al. 2018 [42]																			
Woodward et al. 2016 [43]																			
Xu et al. 2018 [44]																			
Young et al. 2013a [45]	1	1	1	1	1	1	1	1	1										
Young et al. 2013b [46]																			
Young et al. 2013c [47]	1	1	1	0	0	1	1	1	1										
Young et al. 2014a [48]																			
Young et al. 2014b [49]	1	1	1	1	1	1	1	1	1										
Young et al. 2016 [50]																			
Zhang et al. 2011 [51]	NP																		

sensors

MDPI

Article	Semitendinosus	Biceps Femoris	Tensor Fasciae Latae	Rectus Femoris	Vastus Lateralis	Vastus Medialis	Sartorius	Adductor Magnus	Gracilis	Biceps Femoris Long Head	Biceps Femoris Short Head	Ground Electrode	Gluteal Maximus	Gluteal Medius	Iliotibial tract	Medial Gastrocnemius	Tibialis Anterior	Peroneus Longus	Gastrocnemius Lateralis
**Zhang et al. 2013 [52]**	1		1	1	1	1		1		1	1	1							
**Zhang et al. 2019 [53]**																			
**Zhang et al. 2019 [54]**																			
**Zhang et al. 2012 [55]**	1			1	1		1	1		1	1								
**Zheng et al. 2013 [56]**	NP																		
**Zheng et al. 2014 [57]**	NP																		
**Zheng et al. 2016 [58]**																			
**Zheng et al. 2019 [59]**																			
**Zhou et al. 2019 [60]**																			

sensors

MDPI

Supplementary Material 4

1. References

[1] D. Moher, A. Liberati, J. Tetzlaff, et D. G. Altman, « Preferred Reporting Items for Systematic Reviews and Meta-Analyses: The PRISMA Statement », PLoS Med., vol. 6, no 7, p. 7, 2009.

[2] L. M. Kmet, R. C. Lee, L. S. Cook, et Alberta Heritage Foundation for Medical Research, Standard quality assessment criteria for evaluating primary research papers from a variety of fields. Edmonton, Alta.: Alberta Heritage Foundation for Medical Research, 2004.

[3] Q. Ai, Y. Zhang, W. Qi, Q. Liu, et and K. Chen, « Research on Lower Limb Motion Recognition Based on Fusion of sEMG and Accelerometer Signals », Symmetry, vol. 9, no 8, p. 147, août 2017, doi: 10.3390/sym9080147.

[4] J. Beil, I. Ehrenberger, C. Scherer, C. Mandery, et T. Asfour, « Human Motion Classification Based on Multi-Modal Sensor Data for Lower Limb Exoskeletons », in 2018 IEEE/RSJ International Conference on Intelligent Robots and Systems (IROS), Madrid, oct. 2018, p. 5431-5436, doi: 10.1109/IROS.2018.8594110.

[5] Baojun Chen et al., « Locomotion Mode Classification Using a Wearable Capacitive Sensing System », IEEE Trans. Neural Syst. Rehabil. Eng., vol. 21, no 5, p. 744-755, sept. 2013, doi: 10.1109/TNSRE.2013.2262952.

[6] B. Chen, E. Zheng, Q. Wang, et L. Wang, « A new strategy for parameter optimization to improve phase-dependent locomotion mode recognition », Neurocomputing, vol. 149, p. 585-593, févr. 2015, doi: 10.1016/j.neucom.2014.08.016.

[7] B. Chen, X. Wang, Y. Huang, K. Wei, et Q. Wang, « A foot-wearable interface for locomotion mode recognition based on discrete contact force distribution », Mechatronics, vol. 32, p. 12-21, déc. 2015, doi: 10.1016/j.mechatronics.2015.09.002.

[8] Lin Du, Fan Zhang, Ming Liu, et He Huang, « Toward Design of an Environment-Aware Adaptive Locomotion-Mode-Recognition System », IEEE Trans. Biomed. Eng., vol. 59, no 10, p. 2716-2725, oct. 2012, doi: 10.1109/TBME.2012.2208641.

[9] Lin Du, Fan Zhang, Haibo He, et He Huang, « Improving the performance of a neural-machine interface for prosthetic legs using adaptive pattern classifiers », in 2013 35th Annual International Conference of the IEEE Engineering in Medicine and Biology Society (EMBC), Osaka, juill. 2013, p. 1571-1574, doi: 10.1109/EMBC.2013.6609814.

[10] Y. Feng, W. Chen, et Q. Wang, « A strain gauge based locomotion mode recognition method using convolutional neural network », Adv. Robot., vol. 33, no 5, p. 254-263, mars 2019, doi: 10.1080/01691864.2018.1563500.

[11] A. K. Godiyal, M. Mondal, S. D. Joshi, et D. Joshi, « Force Myography Based Novel Strategy for Locomotion Classification », IEEE Trans. Hum.-Mach. Syst., vol. 48, no 6, p. 648-657, déc. 2018, doi: 10.1109/THMS.2018.2860598.

[12] C. Gong, D. Xu, Z. Zhou, N. Vitiello, et Q. Wang, « Real-Time On-Board Recognition of Locomotion Modes for an Active Pelvis Orthosis », in 2018 IEEE-RAS 18th International Conference on Humanoid Robots (Humanoids), Beijing, China, nov. 2018, p. 346-350, doi: 10.1109/HUMANOIDS.2018.8625044.

[13] C. Gong, D. Xu, Z. Zhou, N. Vitiello, et Q. Wang, « BPNN-Based Real-Time Recognition of Locomotion Modes for an Active Pelvis Orthosis with Different Assistive Strategies », Int. J. Humanoid Robot., vol. 17, no 01, p. 2050004, févr. 2020, doi: 10.1142/S0219843620500048.

[14] R. Hernandez, Fan Zhang, Xiaorong Zhang, He Huang, et Qing Yang, « Promise of a low power mobile CPU based embedded system in artificial leg control », in 2012 Annual International Conference of the IEEE Engineering in Medicine and Biology Society, San Diego, CA, août 2012, p. 5250-5253, doi: 10.1109/EMBC.2012.6347178.

[15] R. Hernandez, Qing Yang, He Huang, Fan Zhang, et Xiaorong Zhang, « Design and implementation of a low power mobile CPU based embedded system for artificial leg control », in 2013 35th Annual International Conference of the IEEE Engineering in Medicine and Biology Society (EMBC), Osaka, juill. 2013, p. 5769-5772, doi: 10.1109/EMBC.2013.6610862.

[16] He Huang, T. A. Kuiken, et R. D. Lipschutz, « A Strategy for Identifying Locomotion Modes Using Surface Electromyography », IEEE Trans. Biomed. Eng., vol. 56, no 1, p. 65-73, janv. 2009, doi: 10.1109/TBME.2008.2003293.

[17] H. Huang, F. Zhang, Y. L. Sun, et H. He, « Design of a robust EMG sensing interface for pattern classification », J. Neural Eng., vol. 7, no 5, p. 056005, oct. 2010, doi: 10.1088/1741-2560/7/5/056005.
[18] He Huang, Fan Zhang, L. J. Hargrove, Zhi Dou, D. R. Rogers, et K. B. Englehart, « Continuous Locomotion-Mode Identification for Prosthetic Legs Based on Neuromuscular–Mechanical Fusion », IEEE Trans. Biomed. Eng., vol. 58, no 10, p. 2867-2875, oct. 2011, doi: 10.1109/TBME.2011.2161671.
[19] H. Kim, Y. J. Shin, et J. Kim, « Kinematic-based locomotion mode recognition for power augmentation exoskeleton », Int. J. Adv. Robot. Syst., vol. 14, no 5, p. 172988141773032, sept. 2017, doi: 10.1177/1729881417730321.
[20] M. Liu, D. Wang, et H. Huang, « Development of an Environment-Aware Locomotion Mode Recognition System for Powered Lower Limb Prostheses », IEEE Trans. Neural Syst. Rehabil. Eng., vol. 24, no 4, p. 434-443, avr. 2016, doi: 10.1109/TNSRE.2015.2420539.
[21] Ming Liu, Fan Zhang, et He Huang, « An Adaptive Classification Strategy for Reliable Locomotion Mode Recognition », Sensors, vol. 17, no 9, p. 2020, sept. 2017, doi: 10.3390/s17092020.
[22] Z. Liu, W. Lin, Y. Geng, et P. Yang, « Intent pattern recognition of lower-limb motion based on mechanical sensors », IEEECAA J. Autom. Sin., vol. 4, no 4, p. 651-660, 2017, doi: 10.1109/JAS.2017.7510619.
[23] Y. Long et al., « PSO-SVM-Based Online Locomotion Mode Identification for Rehabilitation Robotic Exoskeletons », Sensors, vol. 16, no 9, p. 1408, sept. 2016, doi: 10.3390/s16091408.
[24] A. Mai et S. Commuri, « Gait identification for an intelligent prosthetic foot », in 2011 IEEE International Symposium on Intelligent Control, Denver, CO, USA, sept. 2011, p. 1341-1346, doi: 10.1109/ISIC.2011.6045418.
[25] J. Mai, D. Xu, H. Li, S. Zhang, J. Tan, et Q. Wang, « Implementing a SoC-FPGA Based Acceleration System for On-Board SVM Training for Robotic Transtibial Prostheses », in 2018 IEEE International Conference on Real-time Computing and Robotics (RCAR), Kandima, Maldives, août 2018, p. 150-155, doi: 10.1109/RCAR.2018.8621732.
[26] J. Mai, W. Chen, S. Zhang, D. Xu, et Q. Wang, « Performance analysis of hardware acceleration for locomotion mode recognition in robotic prosthetic control », in 2018 IEEE International Conference on Cyborg and Bionic Systems (CBS), Shenzhen, oct. 2018, p. 607-611, doi: 10.1109/CBS.2018.8612257.
[27] J. D. Miller, M. S. Beazer, et M. E. Hahn, « Myoelectric Walking Mode Classification for Transtibial Amputees », IEEE Trans. Biomed. Eng., vol. 60, no 10, p. 2745-2750, oct. 2013, doi: 10.1109/TBME.2013.2264466.
[28] D.-H. Moon, D. Kim, et Y.-D. Hong, « Development of a Single Leg Knee Exoskeleton and Sensing Knee Center of Rotation Change for Intention Detection », Sensors, vol. 19, no 18, p. 3960, sept. 2019, doi: 10.3390/s19183960.
[29] C. Pew et G. K. Klute, « Turn Intent Detection For Control of a Lower Limb Prosthesis », IEEE Trans. Biomed. Eng., vol. 65, no 4, p. 789-796, avr. 2018, doi: 10.1109/TBME.2017.2721300.
[30] C. E. Shell, G. K. Klute, et R. R. Neptune, « Identifying classifier input signals to predict a cross-slope during transtibial amputee walking », PLOS ONE, vol. 13, no 2, p. e0192950, févr. 2018, doi: 10.1371/journal.pone.0192950.
[31] A. M. Simon et al., « Delaying Ambulation Mode Transition Decisions Improves Accuracy of a Flexible Control System for Powered Knee-Ankle Prosthesis », IEEE Trans. Neural Syst. Rehabil. Eng., vol. 25, no 8, p. 1164-1171, août 2017, doi: 10.1109/TNSRE.2016.2613020.
[32] J. A. Spanias, E. J. Perreault, et L. J. Hargrove, « A strategy for labeling data for the neural adaptation of a powered lower limb prosthesis », in 2014 36th Annual International Conference of the IEEE Engineering in Medicine and Biology Society, Chicago, IL, août 2014, p. 3090-3093, doi: 10.1109/EMBC.2014.6944276.
[33] J. A. Spanias, A. M. Simon, K. A. Ingraham, et L. J. Hargrove, « Effect of additional mechanical sensor data on an EMG-based pattern recognition system for a powered leg prosthesis », in 2015 7th International IEEE/EMBS Conference on Neural Engineering (NER), Montpellier, France, avr. 2015, p. 639-642, doi: 10.1109/NER.2015.7146704.
[34] J. A. Spanias, E. J. Perreault, et L. J. Hargrove, « Detection of and Compensation for EMG Disturbances for Powered Lower Limb Prosthesis Control », IEEE Trans. Neural Syst. Rehabil. Eng., vol. 24, no 2, p. 226-234, févr. 2016, doi: 10.1109/TNSRE.2015.2413393.
[35] J. A. Spanias, A. M. Simon, E. J. Perreault, et L. J. Hargrove, « Preliminary results for an adaptive pattern recognition system for novel users using a powered lower limb prosthesis », in 2016 38th Annual International Conference of the IEEE Engineering in Medicine and Biology Society (EMBC), Orlando, FL, USA, août 2016, p. 5083-5086, doi: 10.1109/EMBC.2016.7591870.

[36] J. A. Spanias, A. M. Simon, et L. J. Hargrove, « Across-user adaptation for a powered lower limb prosthesis », in 2017 International Conference on Rehabilitation Robotics (ICORR), London, juill. 2017, p. 1580-1583, doi: 10.1109/ICORR.2017.8009473.
[37] J. A. Spanias, A. M. Simon, S. B. Finucane, E. J. Perreault, et L. J. Hargrove, « Online adaptive neural control of a robotic lower limb prosthesis », J. Neural Eng., vol. 15, no 1, p. 016015, févr. 2018, doi: 10.1088/1741-2552/aa92a8.
[38] R. Stolyarov, G. Burnett, et H. Herr, « Translational Motion Tracking of Leg Joints for Enhanced Prediction of Walking Tasks », IEEE Trans. Biomed. Eng., vol. 65, no 4, p. 763-769, avr. 2018, doi: 10.1109/TBME.2017.2718528.
[39] B.-Y. Su, J. Wang, S.-Q. Liu, M. Sheng, J. Jiang, et K. Xiang, « A CNN-Based Method for Intent Recognition Using Inertial Measurement Units and Intelligent Lower Limb Prosthesis », IEEE Trans. Neural Syst. Rehabil. Eng., vol. 27, no 5, p. 1032-1042, mai 2019, doi: 10.1109/TNSRE.2019.2909585.
[40] D. C. Tkach et L. J. Hargrove, « Neuromechanical sensor fusion yields highest accuracies in predicting ambulation mode transitions for trans-tibial amputees », in 2013 35th Annual International Conference of the IEEE Engineering in Medicine and Biology Society (EMBC), Osaka, juill. 2013, p. 3074-3077, doi: 10.1109/EMBC.2013.6610190.
[41] X. Wang, Q. Wang, E. Zheng, K. Wei, et L. Wang, « A Wearable Plantar Pressure Measurement System: Design Specifications and First Experiments with an Amputee », in Intelligent Autonomous Systems 12, vol. 194, S. Lee, H. Cho, K.-J. Yoon, et J. Lee, Éd. Berlin, Heidelberg: Springer Berlin Heidelberg, 2013, p. 273-281.
[42] C. Wang, X. Wu, Y. Ma, G. Wu, et Y. Luo, « A Flexible Lower Extremity Exoskeleton Robot with Deep Locomotion Mode Identification », Complexity, vol. 2018, p. 1-9, oct. 2018, doi: 10.1155/2018/5712108.
[43] R. B. Woodward, J. A. Spanias, et L. J. Hargrove, « User intent prediction with a scaled conjugate gradient trained artificial neural network for lower limb amputees using a powered prosthesis », in 2016 38th Annual International Conference of the IEEE Engineering in Medicine and Biology Society (EMBC), Orlando, FL, USA, août 2016, p. 6405-6408, doi: 10.1109/EMBC.2016.7592194.
[44] D. Xu, Y. Feng, J. Mai, et Q. Wang, « Real-Time On-Board Recognition of Continuous Locomotion Modes for Amputees With Robotic Transtibial Prostheses », IEEE Trans. Neural Syst. Rehabil. Eng., vol. 26, no 10, p. 2015-2025, oct. 2018, doi: 10.1109/TNSRE.2018.2870152.
[45] A. J. Young, A. Simon, et L. J. Hargrove, « An intent recognition strategy for transfemoral amputee ambulation across different locomotion modes », in 2013 35th Annual International Conference of the IEEE Engineering in Medicine and Biology Society (EMBC), Osaka, juill. 2013, p. 1587-1590, doi: 10.1109/EMBC.2013.6609818.
[46] A. J. Young, A. M. Simon, N. P. Fey, et L. J. Hargrove, « Intent Recognition in a Powered Lower Limb Prosthesis Using Time History Information », Ann. Biomed. Eng., vol. 42, no 3, p. 631-641, mars 2014, doi: 10.1007/s10439-013-0909-0.
[47] A. J. Young, A. M. Simon, N. P. Fey, et L. J. Hargrove, « Classifying the intent of novel users during human locomotion using powered lower limb prostheses », in 2013 6th International IEEE/EMBS Conference on Neural Engineering (NER), San Diego, CA, USA, nov. 2013, p. 311-314, doi: 10.1109/NER.2013.6695934.
[48] A. J. Young, A. M. Simon, et L. J. Hargrove, « A Training Method for Locomotion Mode Prediction Using Powered Lower Limb Prostheses », IEEE Trans. Neural Syst. Rehabil. Eng., vol. 22, no 3, p. 671-677, mai 2014, doi: 10.1109/TNSRE.2013.2285101.
[49] A. J. Young, T. A. Kuiken, et L. J. Hargrove, « Analysis of using EMG and mechanical sensors to enhance intent recognition in powered lower limb prostheses », J. Neural Eng., vol. 11, no 5, p. 056021, oct. 2014, doi: 10.1088/1741-2560/11/5/056021.
[50] A. J. Young et L. J. Hargrove, « A Classification Method for User-Independent Intent Recognition for Transfemoral Amputees Using Powered Lower Limb Prostheses », IEEE Trans. Neural Syst. Rehabil. Eng., vol. 24, no 2, p. 217-225, févr. 2016, doi: 10.1109/TNSRE.2015.2412461.
[51] F. Zhang, W. DiSanto, J. Ren, Z. Dou, Q. Yang, et H. Huang, « A Novel CPS System for Evaluating a Neural-Machine Interface for Artificial Legs », in 2011 IEEE/ACM Second International Conference on Cyber-Physical Systems, Chicago, IL, USA, avr. 2011, p. 67-76, doi: 10.1109/ICCPS.2011.13.
[52] Fan Zhang et He Huang, « Source Selection for Real-Time User Intent Recognition Toward Volitional Control of Artificial Legs », IEEE J. Biomed. Health Inform., vol. 17, no 5, p. 907-914, sept. 2013, doi: 10.1109/JBHI.2012.2236563.

[53] K. Zhang, W. Zhang, W. Xiao, H. Liu, C. W. De Silva, et C. Fu, « Sequential Decision Fusion for Environmental Classification in Assistive Walking », IEEE Trans. Neural Syst. Rehabil. Eng., vol. 27, no 9, p. 1780-1790, sept. 2019, doi: 10.1109/TNSRE.2019.2935765.

[54] K. Zhang et al., « Environmental Features Recognition for Lower Limb Prostheses Toward Predictive Walking », IEEE Trans. Neural Syst. Rehabil. Eng., vol. 27, no 3, p. 465-476, mars 2019, doi: 10.1109/TNSRE.2019.2895221.

[55] Xiaorong Zhang, Ding Wang, Qing Yang, et He Huang, « An automatic and user-driven training method for locomotion mode recognition for artificial leg control », in 2012 Annual International Conference of the IEEE Engineering in Medicine and Biology Society, San Diego, CA, août 2012, p. 6116-6119, doi: 10.1109/EMBC.2012.6347389.

[56] Enhao Zheng, Long Wang, Yimin Luo, Kunlin Wei, et Qining Wang, « Non-contact capacitance sensing for continuous locomotion mode recognition: Design specifications and experiments with an amputee », in 2013 IEEE 13th International Conference on Rehabilitation Robotics (ICORR), Seattle, WA, juin 2013, p. 1-6, doi: 10.1109/ICORR.2013.6650410.

[57] Enhao Zheng, Long Wang, Kunlin Wei, et Qining Wang, « A Noncontact Capacitive Sensing System for Recognizing Locomotion Modes of Transtibial Amputees », IEEE Trans. Biomed. Eng., vol. 61, no 12, p. 2911-2920, déc. 2014, doi: 10.1109/TBME.2014.2334316.

[58] E. Zheng et Q. Wang, « Noncontact Capacitive Sensing-Based Locomotion Transition Recognition for Amputees With Robotic Transtibial Prostheses », IEEE Trans. Neural Syst. Rehabil. Eng., vol. 25, no 2, p. 161-170, févr. 2017, doi: 10.1109/TNSRE.2016.2529581.

[59] E. Zheng, Q. Wang, et H. Qiao, « Locomotion Mode Recognition With Robotic Transtibial Prosthesis in Inter-Session and Inter-Day Applications », IEEE Trans. Neural Syst. Rehabil. Eng., vol. 27, no 9, p. 1836-1845, sept. 2019, doi: 10.1109/TNSRE.2019.2934525.

[60] Z. Zhou, X. Liu, Y. Jiang, J. Mai, et Q. Wang, « Real-time onboard SVM-based human locomotion recognition for a bionic knee exoskeleton on different terrains », in 2019 Wearable Robotics Association Conference (WearRAcon), Scottsdale, AZ, USA, mars 2019, p. 34-39, doi: 10.1109/WEARRACON.2019.8719399.
